# Altered gut microbiota and untargeted metabolomic features in patients with schizophrenia: a case–control study

**DOI:** 10.3389/fpsyt.2026.1981601

**Published:** 2026-10-22

**Authors:** Yunqiang Mi, Peng Sun, Qingrui Hao, Yuanjun Yang, Chang Dong, Cunzhe Kong, Liqing Li, Chunhai Zhao, Hong Yin

**Affiliations:** 1 The 984th Hospital of Joint Logistics Support Force of the Chinese People’s Liberation Army, Beijing, China; 2 The 8th Medical Center of the Chinese People’s Liberation Army General Hospital, Beijing, China

**Keywords:** 16S rRNA gene sequencing16S rRNA, gut microbiota, microbiome analysis, microbiota–gut–brain axis, schizophrenia, untargeted metabolomics

## Abstract

**Background:**

Mental disorders are among the leading causes of global disease burden, and their etiology spans genetic, environmental, and physiological factors. Emerging evidence suggests that the gut microbiota may contribute to the development of neuropsychiatric disorders through the microbiota–gut–brain axis. This study aimed to characterize differences in gut microbial composition and untargeted metabolomic profiles between patients with schizophrenia and healthy controls.

**Methods:**

Fecal samples were collected from 30 patients with schizophrenia and 30 healthy controls; 59 samples (30 patients and 29 controls) yielded qualified 16S rRNA gene sequencing data. Untargeted metabolomic profiling was performed by ultra-high-performance liquid chromatography–tandem mass spectrometry (UHPLC-MS/MS).

**Results:**

Compared with healthy controls, patients with schizophrenia showed reduced microbial richness and diversity together with marked separation in β-diversity. In an exploratory random forest ranking, Pseudocitrobacter, Hafnia-Obesumbacterium, and Escherichia-Shigella contributed most strongly to between-group discrimination. Escherichia-Shigella was significantly enriched in patients (median relative abundance 2.377% in group S vs. 0.119% in group H; q = 0.00031; Cliff’s δ = 0.646), whereas Faecalibacterium (2.630% vs. 7.275%) and Agathobacter (0.903% vs. 2.874%) were more abundant in healthy controls. Untargeted metabolomics likewise revealed between-group differences that predominantly involved metabolites and pathways of amino acid and lipid metabolism.

**Conclusion:**

Patients with schizophrenia show alterations in gut microbial composition and untargeted metabolomic features, supporting an association between schizophrenia and microbiota–metabolite alterations. Future confirmation requires larger longitudinal cohorts and independent validation datasets. Owing to the small sample size, cross-sectional study design, and antipsychotic drug exposure, we could not disentangle disease-specific effects from medication-related effects.

## Introduction

According to a World Health Organization report, nearly one billion people worldwide were living with a mental disorder in 2019. Mental disorders impair everyday functioning, particularly the ability to fulfill social roles. This impairment is especially pronounced in mood disorders; in psychotic disorders and autism spectrum disorder (ASD), disturbances of emotion, cognition, and communication translate directly into severe limitations in physical, mental, and social functioning (1). Mental disorders are complex and multifactorial, and accumulating evidence indicates that the gut and the brain communicate bidirectionally (2).

This bidirectional communication is mediated by neural, endocrine, and immune signals (3). The gut microbiome is now recognized as a potentially important modifier of neurological function across the human lifespan (4) and is thought to influence neurotransmitter synthesis, myelination of prefrontal cortical neurons, and the development of the amygdala and hippocampus (5). Numerous studies have reported gut microbial alterations in depression, anxiety, ASD, and schizophrenia (5–9). Interactions between the microbiota and the central nervous system are mediated mainly by neural (autonomic), hormonal (hypothalamic–pituitary–adrenal axis), and immune (cytokine and chemokine) routes, which are closely interconnected (10). Collectively, these pathways constitute the microbiota–gut–brain axis (11).

Peripheral factors that regulate the central nervous system are attracting growing attention, including the mechanisms by which endocrine and immune signals modulate reward processing. Alterations in the gut microbiome have been detected at several taxonomic levels in fecal samples from patients with metabolic and autoimmune diseases (12), and many studies have linked psychiatric disorders to shifts in the composition of the fecal microbiota (13). Of note, neurological insults such as traumatic brain injury (TBI) can activate the hypothalamic–pituitary–adrenal (HPA) axis and the sympathetic nervous system, raising cortisol and catecholamine levels (14); elevated cortisol, in turn, increases intestinal barrier permeability (15).

Most existing studies have focused on a single omics layer, and few investigations, particularly from China, have integrated fecal microbiota profiling with untargeted metabolomics. To help address this gap, we simultaneously assessed microbial taxonomic features and untargeted metabolomic features and applied an integrated multi-omics approach to explore how gut microbial shifts relate to host metabolic characteristics. Our aim was to identify microbiota–metabolite associations in schizophrenia and to provide a basis for future mechanistic and clinical studies.

## Materials and methods

### Sample collection

Fecal samples were collected from 60 participants: 30 healthy controls (group H) and 30 patients with schizophrenia (group S). Of these, 59 samples yielded qualified 16S rRNA gene sequencing data and were included in the microbiota analysis (30 from group S and 29 from group H; one sample from group H produced no usable data). Controls were healthy individuals without acute or chronic disease who underwent routine health check-ups at the physical examination center of our hospital; trained psychiatrists confirmed the absence of current or past mental disorders using the Structured Clinical Interview for DSM-5 (SCID-5). Patients met DSM-5 criteria for schizophrenia, were hospitalized for the first time, and had received psychotropic medication for no more than 30 days. Diagnoses were established independently by at least two trained psychiatrists using SCID-5. All materials analyzed consisted of residual specimens remaining after routine clinical stool examination (collected as part of inpatient work-ups for patients and routine health check-up packages for controls); no additional sampling was performed for research purposes.

No participant had taken probiotics, prebiotics, laxatives, or antibiotics within the month before sampling. Fresh fecal samples were stored immediately at −80 °C until analysis, and fasting blood samples were collected for serum biochemical testing. Individuals with gastrointestinal, metabolic, inflammatory, or autoimmune disease, or with a recent infection, were excluded. At the time of sampling, information on body mass index (BMI), smoking, alcohol consumption, bowel habits, and dietary composition was recorded.

This exploratory case–control study did not include a prospective sample-size calculation. Given the substantial inter-individual variation of the gut microbiome, the statistical power of a sample of this size (29 vs. 30) is limited; the findings are therefore intended primarily to generate hypotheses for validation in larger cohorts.

### DNA extraction and 16S rRNA gene sequencing

Microbial DNA was extracted from fecal samples using the E.Z.N.A.^®^ Stool DNA Kit (catalog no. D4015, Omega Bio-tek, USA) according to the manufacturer’s instructions. The V3–V4 variable regions of the bacterial 16S rRNA gene were amplified by PCR under the following conditions: initial denaturation at 95 °C for 3 min; 25 cycles of denaturation at 95 °C for 30 s, annealing at 55 °C for 30 s, and extension at 72 °C for 30 s; and a final extension at 72 °C for 5 min. Amplification was performed with primers 338F (5′-ACTCCTACGGGAGGCAGCAG-3′) and 806R (5′-GGACTACHVGGGTWTCTAAT-3′), each bearing a unique 8-bp barcode for sample identification after pooling.

Each PCR was carried out in triplicate in a total volume of 20 μL containing 4 μL of 5× FastPfu buffer, 2 μL of 2.5 mM dNTPs, 0.8 μL of each primer (5 μM), 0.4 μL of FastPfu polymerase, and 10 ng of template DNA. Purified amplicons were pooled in equimolar amounts and sequenced on an Illumina NovaSeq 6000 platform (Illumina, San Diego, CA, USA) in 2 × 251 bp paired-end mode, following the standard operating procedure of Shanghai Majorbio Bio-Pharm Technology Co., Ltd.

### Sequencing data processing

Primer sequences were trimmed from paired-end reads in anchored mode using cutadapt (version 5.2), and read pairs that did not match the primers were discarded. Trimmed read pairs were merged with vsearch (version 2.32.0; minimum overlap 20 bp, maximum 5 mismatches) and quality-filtered (maximum expected error maxEE ≤ 1.0; length 350–550 bp). Merged sequences were dereplicated, and noise was removed with the UNOISE3 algorithm to generate amplicon sequence variants (ASVs; minimum abundance threshold, 8 reads). Chimeras were predicted and removed using the uchime3 *de novo* algorithm, and high-quality reads were mapped to ASV sequences to build an ASV abundance table. ASVs were taxonomically classified with the vsearch sintax classifier against the SILVA v138.1 reference database (NR99 training set), using a genus-level confidence threshold of 0.8. All steps used a fixed random seed, and software versions (cutadapt 5.2; vsearch 2.32.0) were recorded to ensure reproducibility. This pipeline replaced the QIIME 2/DADA2 workflow (16) used in the initial analysis of these data, so that every result reported here derives from a single, fully scripted pipeline. Two limitations of this approach should be noted. First, as only the V3–V4 hypervariable region of the 16S rRNA gene was amplified, taxonomic assignment was limited to the genus level without species-level resolution. Genera such as Escherichia-Shigella encompass both pathogenic and nonpathogenic species that cannot be distinguished via 16S rRNA sequencing. Second, 16S rRNA profiling yields community composition only and provides no direct functional information, so any functional inference requires confirmation by shotgun metagenomics.

### Bioinformatics analysis

Within-sample richness and diversity (collectively, α-diversity) were quantified using complementary indices: Chao1, ACE, and Sobs reflect taxon richness, whereas the Shannon and inverse Simpson indices reflect diversity and evenness. β-diversity was used to assess between-group differences in community composition: Bray–Curtis dissimilarity was calculated from ASV relative abundances and Jaccard dissimilarity from ASV presence/absence, and both were visualized by principal coordinate analysis (PCoA). Permutational multivariate analysis of variance (PERMANOVA) with 9,999 label permutations was applied to each distance matrix to test overall between-group compositional differences, and pseudo-F, R^2^, and p-values were reported. Differential abundance testing was performed at genus level on the 59 genera with a mean relative abundance greater than or equal to 0.1% in either group that were detected in at least 6 samples. The threshold of greater than or equal to 0.1% represents a widely used low-abundance filter to remove noise, while requiring detection in at least six samples (approximately 20% of the cohort) retains reliably detectable and biologically interpretable taxa. We chose the Wilcoxon rank-sum test rather than linear discriminant analysis effect size (LEfSe). LEfSe scores tend to be unstable for small-sample datasets. By contrast, the Wilcoxon rank-sum test combined with false-discovery-rate correction and explicit effect-size reporting offers greater analytical transparency. p-values were corrected for multiple comparisons by the Benjamini–Hochberg method (q < 0.05 considered significant), and effect sizes (Cliff’s delta and log2 fold change) were reported. α-diversity indices were calculated and compared in Python (v3.8) using the Wilcoxon rank-sum test, supplemented by Welch’s t test and the Shapiro–Wilk normality test to assess the robustness of the conclusions.

### Exploratory random forest variable importance analysis

We performed the random forest analysis to rank the microbial taxa contributing most to between-group discrimination and to assess their relative importance; it was not intended to build a diagnostic or prognostic classifier. The model was trained on genus-level relative abundances. Features comprised 98 genera with a mean relative abundance greater than or equal to 0.01% and detected in at least 20% of samples. Analysis was performed in Python (v3.8) using the RandomForestClassifier implementation in scikit-learn, with 1,000 trees (n_estimators = 1000) and setting max_features—the number of variables considered at each split—to p/3, where p is the total number of feature genera. Variable importance was measured as Gini importance (mean decrease in impurity).

Discriminative performance was evaluated by stratified five-fold cross-validation: the 59 samples were partitioned into five folds stratified by group (H vs. S), the model was trained on four folds in each round, and the area under the receiver operating characteristic curve (AUC) was computed on the held-out fold; results were then averaged across the five folds. The procedure was repeated 20 times to assess stability. No feature pre-screening based on between-group differences was applied within cross-validation, so as to avoid data snooping and overfitting. The cross-validated AUC for discriminating group H from group S was 0.989 (95% CI 0.970–1.000), and the mean AUC across the 20 repeats was 0.984 (SD 0.009). Two caveats apply. First, because no independent external validation cohort was available, this AUC reflects only within-sample discriminative performance; it is likely optimistically biased and cannot be extrapolated to clinical diagnosis. Second, the variable importance ranking is likewise sample-specific and should not be interpreted as a stable biomarker panel.

### UHPLC-MS/MS analysis

Untargeted metabolomic profiling was performed by ultra-high-performance liquid chromatography–tandem mass spectrometry (UHPLC-MS/MS) on a Vanquish UHPLC system (Thermo Fisher Scientific, Germany) coupled to an Orbitrap Q Exactive HF mass spectrometer (Thermo Fisher Scientific, Germany). Samples were injected onto a Hypersil Gold column (100 mm × 2.1 mm, 1.9 μm) and separated by gradient elution at a flow rate of 0.2 mL/min. In positive ion mode, the mobile phases were solvent A (water containing 0.1% formic acid) and solvent B (methanol); in negative ion mode, they were solvent A (5 mM ammonium acetate, pH 9.0) and solvent B (methanol). The gradient program was as follows: 2% B for 1.5 min, increased to 85% B by 3 min and to 100% B by 10 min, returned to 2% B at 10.1 min, and held at 2% B until 12 min. The mass spectrometer was operated in both positive and negative polarity with the following settings: spray voltage 3.5 kV, capillary temperature 320 °C, sheath gas flow 35 arb, auxiliary gas flow 10 arb, S-lens RF level 60, and auxiliary gas heater temperature 350 °C.

### Data processing and putative metabolite annotation

We processed raw UHPLC-MS/MS data in Compound Discoverer 3.1 (Thermo Fisher Scientific) for peak alignment, peak detection, and relative quantification of metabolite features. The main settings were a retention time window of 0.2 min, mass accuracy of 5 ppm, intensity tolerance of 30%, and a signal-to-noise ratio threshold of 3, with additional intensity-based criteria applied as recommended by the software. Peak intensities were normalized to total spectral intensity to ensure comparability across samples, and molecular formulas were predicted from adduct ions, molecular ion peaks, and fragment ions. The resulting features were matched against the mzCloud, mzVault, and MassList databases to obtain putative metabolite annotations and relative abundance profiles. These annotations were derived solely from database matching and were not confirmed with authentic reference standards; they are therefore putative, and their accuracy requires targeted validation.

### Statistical analysis

Continuous variables were tested for normality with the Shapiro–Wilk test and then compared between groups using the independent-samples Wilcoxon rank-sum test or Student’s t test, as appropriate; categorical variables were compared using Fisher’s exact test or the chi-square test, as appropriate. Statistical analyses for putatively annotated differential metabolites were performed using R (v4.0.3) and Python (v3.8) running on a CentOS 6.6 platform. Note that this CentOS release is no longer officially supported; raw analysis scripts have been preserved to ensure analytical reproducibility. Before analysis, metabolite peak intensities were normalized to relative peak areas, and metabolites with a coefficient of variation (CV) exceeding 30% in quality control (QC) samples were excluded. In both the metabolomic and the microbial differential abundance analyses, p-values were corrected for multiple comparisons using the Benjamini–Hochberg false discovery rate (FDR) method, with an FDR-corrected q < 0.05 considered statistically significant.

Spearman rank correlation analysis was used to assess associations between differentially abundant genera and differential metabolites. Genus-level relative abundances were obtained by aggregating the ASVs generated in the UNOISE3 re-analysis described above. The resulting p-values were corrected for multiple testing by the Benjamini–Hochberg method, with corrected q < 0.05 considered statistically significant. Heatmaps were generated in R. Metabolite annotation information was retrieved from multiple databases, including the Kyoto Encyclopedia of Genes and Genomes (KEGG) (17), the Human Metabolome Database (HMDB), and LIPID MAPS.

## Results

### Participant characteristics

The study sample comprised 60 individuals: 30 patients with schizophrenia (group S) and 30 healthy individuals (group H). Fifty-nine samples (30 from group S and 29 from group H) yielded usable 16S rRNA gene sequencing data and were included in the microbiota analysis. Demographic and clinical characteristics are summarized in **Table 1**. The groups did not differ significantly in age or sex, but neutrophil count, red blood cell (RBC) count, estradiol (E2), and prolactin (PRL) did differ significantly; PRL and E2 were markedly higher in group S than in group H. Two important caveats should be noted. First, although data regarding BMI, smoking status, alcohol intake, bowel habits and dietary patterns were recorded at sampling, these datasets remained incomplete. Accordingly, they were neither presented in **Table 1** nor compared across groups, which limits our ability to evaluate baseline group balance. Diet is a major determinant of gut microbial composition, and bowel habits also substantially influence fecal microbiota profiles; neither was matched between groups nor adjusted for statistically. Second, the between-group differences in PRL, E2, and neutrophil count indicate that these variables should be treated as potential confounders when interpreting the microbiota and metabolomic findings.

**Table 1:** Demographic and clinical characteristics of patients with schizophrenia and healthy controls (clinical indicators are based on all 60 included participants; of these, 59 completed 16S rRNA gene sequencing and were included in the microbiota analysis).

Variable	Healthy control, H (n = 30)	Schizophrenia, S (n = 30)	p-value
Sex, n (%)			0.405
Female	11 (36.7%)	8 (26.7%)	
Male	19 (63.3%)	22 (73.3%)	
Age, years	34.50 (29.25–40.75)	33.50 (26.00–45.25)	0.684
WBC, ×10^9^/L	5.93 ± 1.51	6.62 ± 1.49	0.078
NEU#, ×10^9^/L	3.15 (2.35–4.08)	3.85 (3.03–4.58)	**0.039**
LYM#, ×10^9^/L	1.95 (1.73–2.40)	1.90 (1.70–2.25)	0.509
RBC, ×10^12^/L	5.00 ± 0.49	4.75 ± 0.45	**0.042**
HGB, g/L	140.87 ± 20.43	139.60 ± 12.52	0.773
PLT, ×10^9^/L	244.10 ± 42.55	228.13 ± 58.01	0.229
ALT, U/L	17.00 (14.00–25.50)	20.50 (13.25–43.00)	0.228
AST, U/L	19.00 (17.25–22.00)	20.50 (16.25–27.00)	0.538
GGT, U/L	17.00 (13.00–22.00)	19.00 (17.00–35.00)	0.111
BUN, mmol/L	4.90 (3.83–5.68)	4.85 (3.93–5.55)	0.790
CREA, μmol/L	76.00 (60.50–87.00)	73.00 (64.75–78.00)	0.359
GLU, mmol/L	4.80 (4.30–5.18)	4.90 (4.40–5.63)	0.185
TESTO, ng/mL	3.47 (0.59–5.27)	3.38 (0.87–5.38)	0.935
PROG, ng/mL	0.66 (0.57–0.83)	0.54 (0.29–1.07)	0.147
PRL, μIU/mL	191.01 (134.20–263.52)	686.05 (406.25–1014.80)	**<0.001**
E2, pg/mL	37.18 (29.08–42.62)	140.50 (71.10–169.00)	**<0.001**

Data are presented as mean ± SD, median (interquartile range, IQR), or n (%), as appropriate. Sex was compared using Pearson’s chi-square test. WBC, RBC, and PLT were compared using Student’s t test; HGB was compared using Welch’s t test. The remaining continuous variables were compared using the Wilcoxon rank-sum test. Bold values indicate statistically significant differences (p < 0.05). ALT, alanine aminotransferase; AST, aspartate aminotransferase; BUN, blood urea nitrogen; CREA, creatinine; E2, estradiol; GGT, gamma-glutamyl transferase; GLU, fasting glucose; HGB, hemoglobin; LYM#, absolute lymphocyte count; NEU#, absolute neutrophil count; PLT, platelet count; PRL, prolactin; PROG, progesterone; RBC, red blood cell count; TESTO, testosterone; WBC, white blood cell count.

### Gut microbiota α-diversity and β-diversity analysis

As shown in **Figures 1A–F**, α-diversity was consistently higher in group H than in group S (all p < 0.01): Sobs (median 4986 vs. 4101), Chao1 (6824 vs. 5533), ACE (6561 vs. 5320), Shannon (5.96 vs. 4.99), and inverse Simpson (48.1 vs. 28.6), indicating reduced richness and diversity of the gut microbiota in patients with schizophrenia. Good’s coverage exceeded 0.97 in both groups, indicating adequate sequencing depth; median coverage was slightly higher in group S than in group H (0.981 vs. 0.978, p = 0.050), most likely reflecting the lower diversity and smaller pool of rare taxa in patients. To compare overall community structure, PCoA was performed on Bray–Curtis distances (**Figure 1G**); the first two axes accounted for 13.3% and 7.8% of the variance, respectively, and the two groups separated clearly. PERMANOVA based on Bray–Curtis distance (9,999 permutations) confirmed a significant difference in community composition between groups (pseudo-F = 3.221, R^2^ = 0.053, p = 0.0001), and the Jaccard-based test was consistent (pseudo-F = 2.924, R^2^ = 0.049, p = 0.0001).

**Figure 1:**
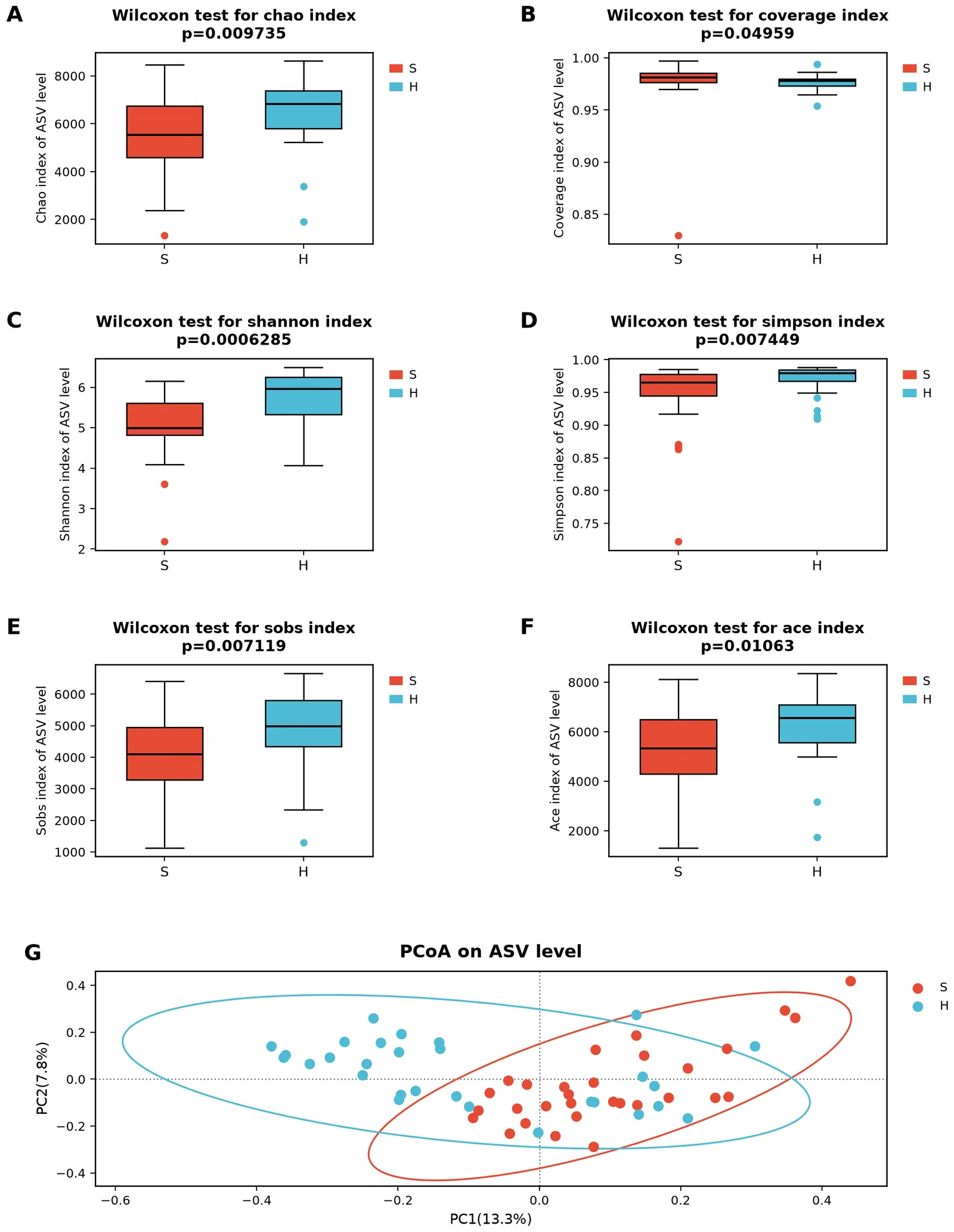
α- and β-diversity of the gut microbiota in patients with schizophrenia and healthy controls (re-analysis of 16S rRNA gene sequencing data). **(A)** Chao1 index; **(B)** Good’s coverage; **(C)** Shannon index; **(D)** Simpson index; **(E)** Sobs index (number of observed ASVs); **(F)** ACE index; **(G)** principal coordinate analysis (PCoA) based on Bray–Curtis distance. Box plots show the median, interquartile range, and full range, with outliers shown as individual dots; group H = healthy controls (n = 29), group S = patients with schizophrenia (n = 30). Between-group comparisons of α-diversity indices were performed using the Wilcoxon rank-sum test (corresponding p-values are shown in each panel title); overall differences in microbial community composition were assessed by PERMANOVA with 9,999 permutations (Bray–Curtis: pseudo-F = 3.221, R^2^ = 0.053, p = 0.0001). Two-sided p < 0.05 was considered statistically significant.

### Gut microbiota composition

Gut microbiota composition differed between patients with schizophrenia and healthy individuals. After denoising, 13,779 ASVs were obtained, of which 13,751 were detected in group H and 13,707 in group S; 13,679 ASVs were shared by the two groups, whereas 72 were unique to group H and 28 to group S (**Figure 2A**). The Venn diagram is provided for descriptive purposes only: these counts are highly sensitive to sequencing depth, and group-specific ASVs mostly correspond to low-abundance rare sequences; hence, no statistical inferences were drawn from this Venn-diagram output.

**Figure 2:**
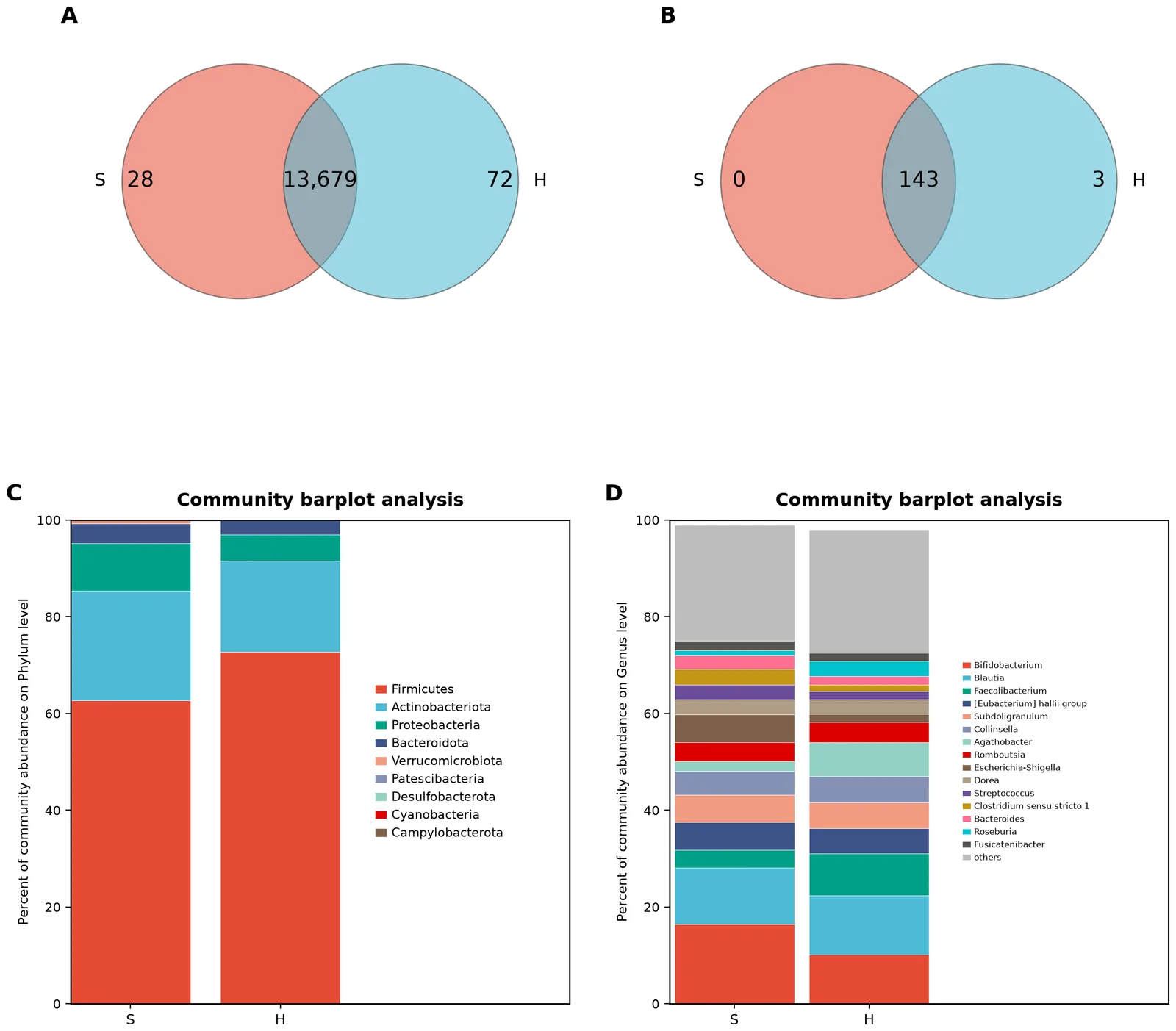
Gut microbiota composition in groups H and S (re-analysis of 16S rRNA gene sequencing data). **(A)** Venn diagram of ASVs shared by, or unique to, groups H and S; **(B)** Venn diagram of genera shared by, or unique to, groups H and S (restricted to taxa assigned to genus level in the SILVA database); **(C)** phylum-level relative abundance (top 15 phyla by mean relative abundance; all remaining taxa grouped as “others”); **(D)** genus-level relative abundance (top 15 genera by mean relative abundance; all remaining taxa grouped as “others”; sequences not assigned to genus level were excluded). At the ASV level, 13,751 and 13,707 ASVs were detected in groups H and S, respectively, of which 13,679 were shared; at the genus level (excluding unassigned taxa), 146 and 143 genera were detected in groups H and S, respectively, of which 143 were shared. Group H = healthy controls; group S = patients with schizophrenia.

At the genus level, 143 genera were detected in group S and 146 in group H, of which 143 were shared by both groups (**Figure 2B**), indicating broadly comparable genus richness. At the phylum level, Firmicutes dominated both groups (median relative abundance 76.3% in group H and 61.1% in group S), followed by Actinobacteriota (16.3% and 18.6%); the median abundance of Proteobacteria was higher in group S than in group H (5.2% vs. 1.4%). Genus-level profiles likewise revealed differences in taxa such as Agathobacter, Faecalibacterium, and Escherichia-Shigella, indicating that relative abundances differed between the two groups at both phylum and genus levels (**Figures 2C, D**).

### Identification of differential microbial taxa

To identify taxa associated with schizophrenia, we compared the abundances of the 59 genera examined (mean relative abundance greater than or equal to 0.1% in either group and detected in at least six samples) between groups S and H using the Wilcoxon rank-sum test, with Benjamini–Hochberg correction (q < 0.05) and reporting of effect sizes (Cliff’s delta and log2 fold change). Twenty-one taxa were differentially abundant: 4 were enriched in group S (Escherichia-Shigella, Terrisporobacter, Klebsiella, and Sarcina) and 17 in group H (16 assigned genera and one group of unassigned ASVs). The genus most strongly enriched in patients was Escherichia-Shigella (median 2.377% [IQR 0.664–5.205] in group S vs. 0.119% [0.035–0.200] in group H; q = 0.00031; Cliff’s delta = 0.646; log2 fold change = 1.77). Genera enriched in controls were predominantly Lachnospiraceae (e.g., Lachnospiraceae NK4A136 group and Lachnospiraceae UCG-008), together with low-abundance genera including Pseudocitrobacter, Raoultella, and Hafnia-Obesumbacterium (**Figures 3A, D**).

**Figure 3:**
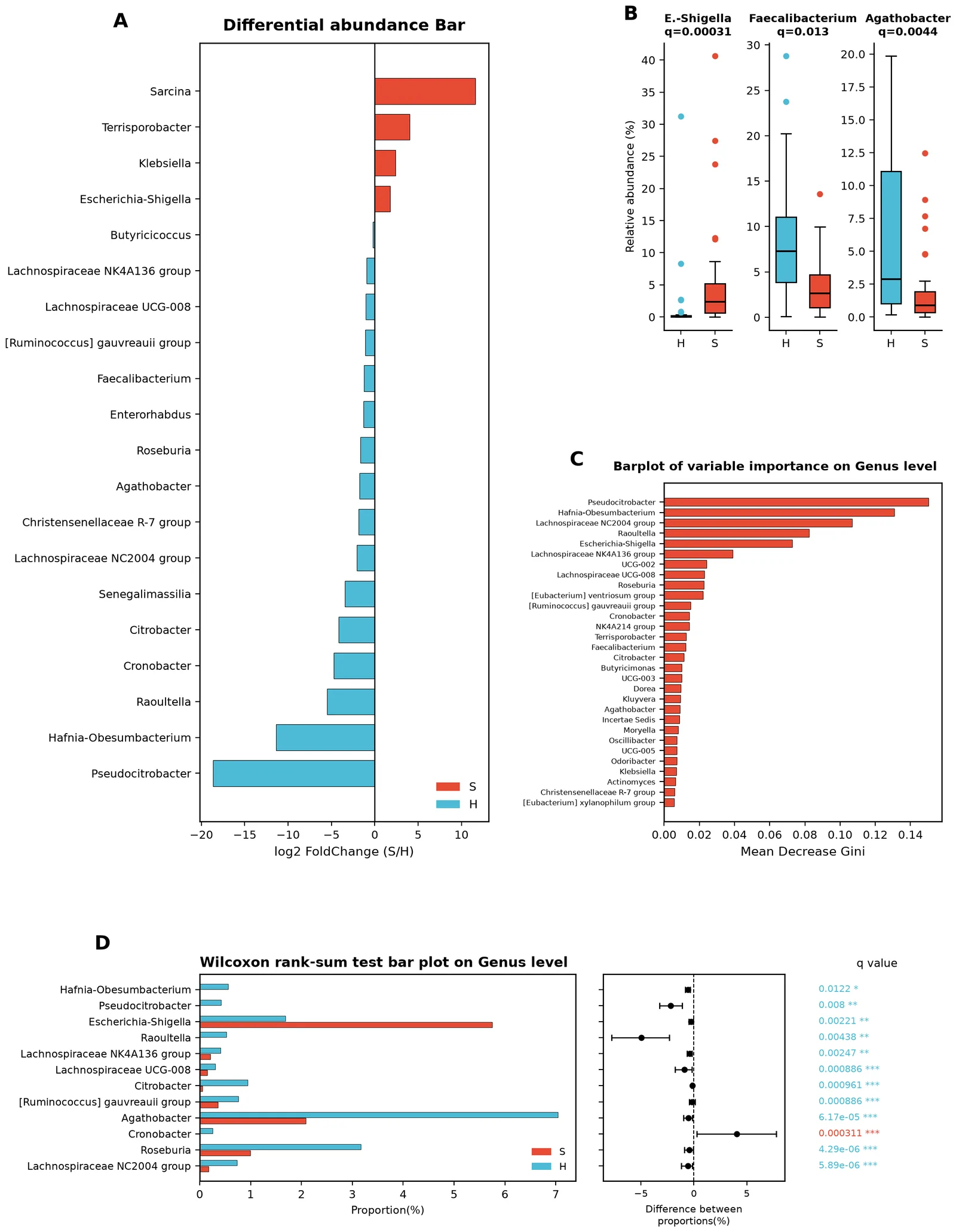
Differentially abundant genera between groups H and S (re-analysis of 16S rRNA gene sequencing data). **(A)** Log2 fold change (S vs. H) for all differentially abundant genera (Wilcoxon q < 0.05; red, enriched in group S; blue, enriched in group H); **(B)** box plots of the relative abundances of key genera (Escherichia-Shigella, Faecalibacterium, and Agathobacter; blue = group H, red = group S); **(C)** top 30 genera by random forest Gini importance; **(D)** bar plot of Wilcoxon rank-sum test results for the 12 genera showing the largest differences (left, mean relative abundance in each group; middle, between-group difference in proportion with 95% bootstrap confidence interval; right, Benjamini–Hochberg-corrected q-value). Group H = healthy controls; group S = patients with schizophrenia. The significance threshold was a Benjamini–Hochberg-corrected q < 0.05.

Random forest Gini importance ranked the genera contributing most to discrimination between groups H and S as Pseudocitrobacter, Hafnia-Obesumbacterium, Lachnospiraceae NC2004 group, Raoultella, and Escherichia-Shigella (**Figure 3C**); the stratified five-fold cross-validated AUC was 0.989. Between-group comparisons of the key genera (**Figures 3B, D**) showed that Escherichia-Shigella was significantly enriched in group S (Wilcoxon U = 716, p = 0.000021, q = 0.00031, log2FC = +1.77); that Faecalibacterium was significantly enriched in group H (median 2.630% [IQR 1.084–4.701] in group S vs. 7.275% [3.861–11.029] in group H; Wilcoxon U = 238, p = 0.0029, q = 0.013, log2FC = −1.26, Cliff’s delta = −0.453); and that Agathobacter was significantly enriched in group H (0.903% [0.347–1.923] in group S vs. 2.874% [1.025–11.077] in group H; Wilcoxon U = 212, p = 0.00074, q = 0.0044, log2FC = −1.76, Cliff’s delta = −0.513). Complete effect-size statistics for all 21 differentially abundant genera—including medians, IQRs, log2FC, Wilcoxon U, p, q, and Cliff’s delta for both groups—are provided in **Supplementary Table S1**. After exclusion of sample S13, which failed quality control, the direction and significance of these differences were unchanged. Within this sample, therefore, Escherichia-Shigella emerged as the key genus enriched in patients, whereas Faecalibacterium and Agathobacter were the key genera enriched in controls. Some genera with high discriminatory importance, such as Hafnia-Obesumbacterium, occurred at extremely low abundance (median 0.022% in group H and 0.0002% in group S), and the direction of their between-group difference was not stable across analytical pipelines; these are therefore described only in exploratory terms. Finally, because of the limited resolution of 16S rRNA gene sequencing, these genus-level differences cannot resolve species-level differences and cannot be used to infer the pathogenicity of the detected genera.

### Metabolomic differences between groups H and S

To identify metabolic alterations associated with schizophrenia, untargeted metabolomic analysis was performed. Both the PCA score plot (**Figure 4A**) and the partial least squares discriminant analysis (PLS-DA) score plot (**Figure 4B**) showed separation between groups H and S, indicating that the overall metabolome differed between the groups. The PLS-DA model showed good explanatory and predictive performance (R^2^X = 0.318, R^2^Y = 0.782, Q^2^ = 0.526), and permutation testing gave no evidence that the model resulted from overfitting (p = 0.005).

**Figure 4:**
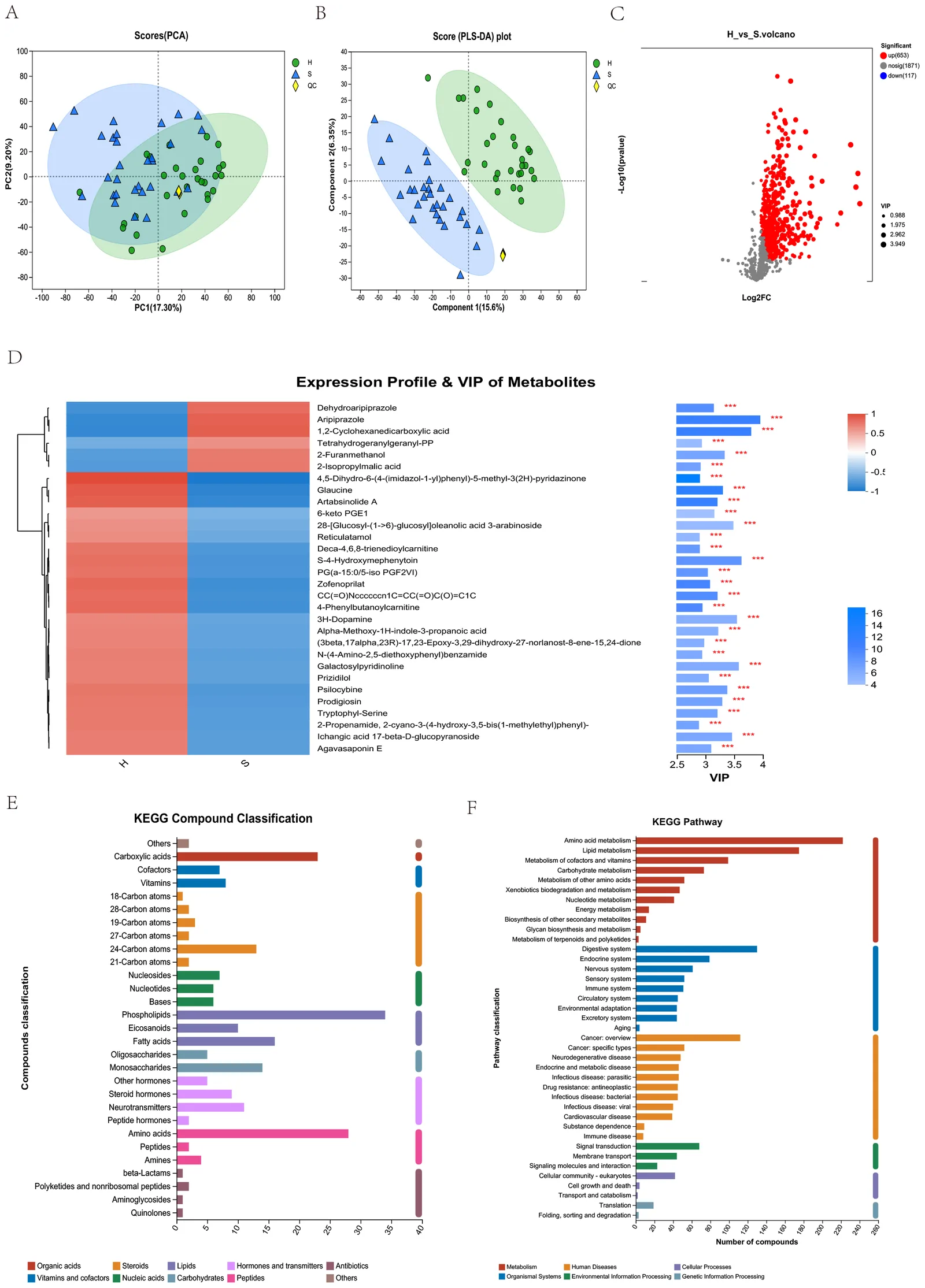
Untargeted metabolomic analysis in patients with schizophrenia and healthy controls. **(A)** PCA score plot showing the overall distribution of metabolomic features in groups H and S; **(B)** PLS-DA score plot showing between-group differences in metabolite profiles; **(C)** volcano plot of differential metabolites between groups H and S; **(D)** heatmap of relative abundances and VIP values of differential metabolites between groups H and S; **(E)** KEGG metabolite compound classification; **(F)** KEGG pathway classification and enrichment analysis of differential metabolites. Group H = healthy controls; group S = patients with schizophrenia. Differential metabolites were provisionally defined as VIP > 1.0, fold change > 1.20 or < 0.83, and FDR-corrected q < 0.05. PLS-DA model parameters: R^2^X = 0.318, R^2^Y = 0.782, Q^2^ = 0.526; permutation test p = 0.005.

The volcano plot shows metabolites with altered abundance in group S relative to group H (**Figure 4C**). Differential metabolites were provisionally defined by combining the following criteria: VIP greater than 1.0, fold change above 1.20 or below 0.83, and FDR-corrected q-value less than 0.05. This combination is standard practice in untargeted metabolomics: it retains metabolites that are statistically significant and show at least a moderate effect size while controlling the false positive rate.

Variable Importance in Projection (VIP) values were derived from the PLS-DA model, and a heatmap was generated to visualize between-group differences in metabolite relative abundances; the two groups were clearly differentiated (**Figure 4D**). According to the KEGG compound classification, the most abundant compounds were carboxylic acids, phospholipids, and amino acids (**Figure 4E**), suggesting that the detected metabolites participate largely in core processes such as energy metabolism and biosynthesis. KEGG pathway enrichment indicated that the dominant functional modules were metabolic pathways, predominantly amino acid and lipid metabolism (**Figure 4F**). Because this analysis relied on database-derived putative annotations that were not confirmed with authentic reference standards, the pathway enrichment results are exploratory.

To explore cross-omics associations, Spearman rank correlation analysis was performed between 18 differentially abundant genera and 30 differential metabolites (VIP greater than 1.0 and FDR q less than 0.05) (**Figure 5**). After Benjamini–Hochberg correction, 312 genus–metabolite correlations reached q < 0.05. Correlation directions followed a clear group-specific pattern. Most genera enriched in the patient group were negatively correlated with the differential metabolites, with Escherichia-Shigella forming the hub of this negative correlation network, showing strong negative correlations with metabolites such as psilocybine (ρ = −0.628), 4,5-dihydro-6-(4-(imidazol-1-yl)phenyl)-5-methyl-3(2H)-pyridazinone (ρ = −0.614), and PG(a-15:0/5-iso PGF2VI) (ρ = −0.528). In contrast, short-chain fatty acid (SCFA)-producing genera enriched in the control group were predominantly positively correlated, with Roseburia, Agathobacter, and Lachnospiraceae UCG-008 showing strong positive correlations with metabolites such as PG(a-15:0/5-iso PGF2VI) (ρ = +0.58 to +0.64) and prizidilol (ρ = +0.59 to +0.60). Two of the features showing the strongest correlations, psilocybine (HMDB0256899) and prizidilol (HMDB0256780), are annotated in the Human Metabolome Database as exogenous compounds belonging to the human exposome rather than as endogenous metabolites. The pyridazinone feature listed above is likewise a drug-like xenobiotic structure. These annotations are therefore unlikely to represent endogenous host metabolism and may instead reflect undocumented dietary, environmental, or medication exposure, or database mis-assignment. They are reported for completeness but are not interpreted as evidence of endogenous metabolic disturbance, and the correlations involving them should be regarded as low confidence. With this caveat, the remaining findings suggest that shifts in microbial community structure and in the metabolite profile are interrelated. Importantly, aripiprazole and its metabolites were significantly negatively correlated with several control-enriched genera (Pseudocitrobacter, Raoultella, and Hafnia-Obesumbacterium) (ρ = −0.43 to −0.49), suggesting that some of the significant correlations reflect drug-related metabolites present only in the patient group; these observations therefore do not support causal inference.

**Figure 5:**
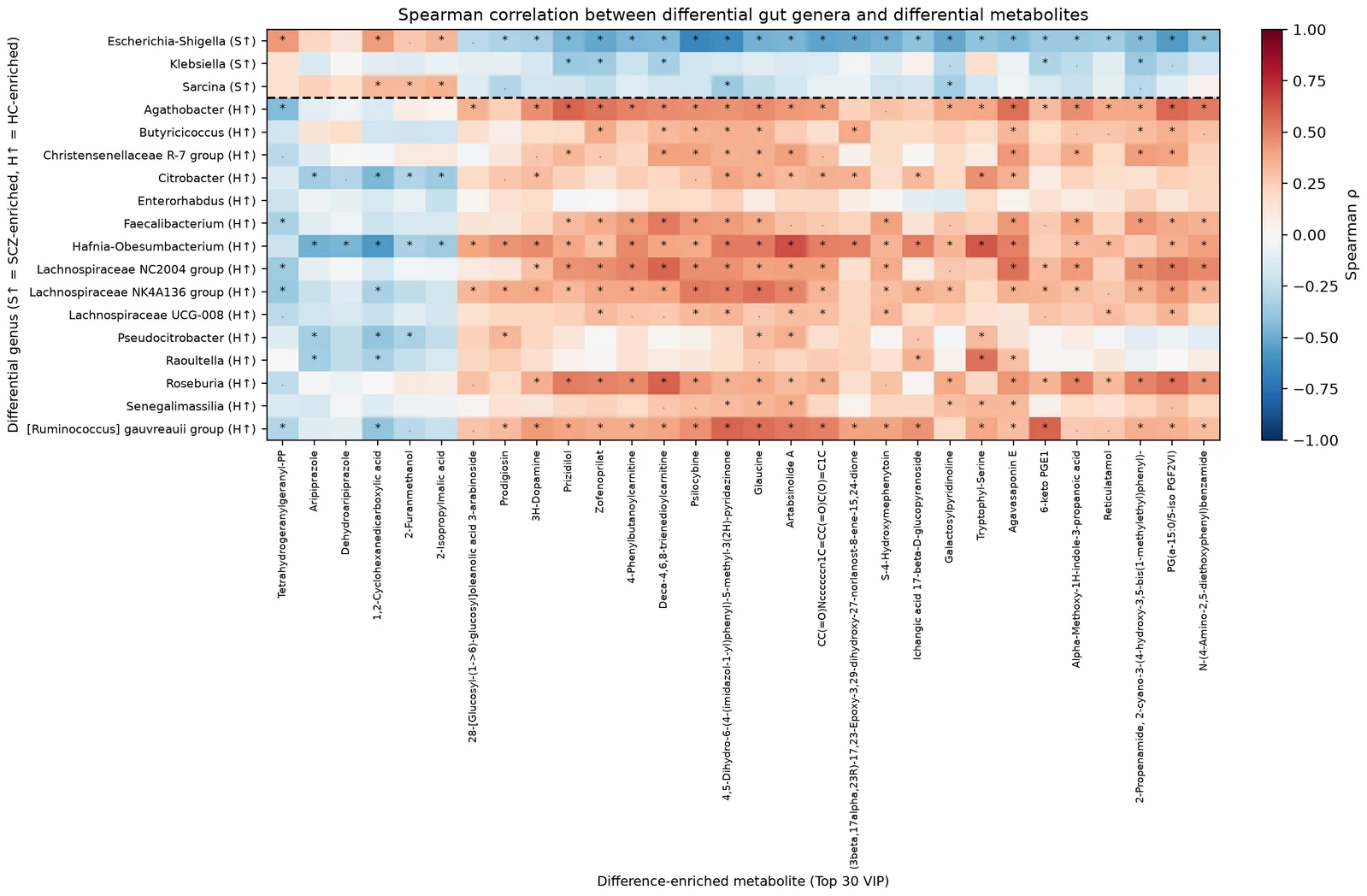
Heatmap of Spearman correlations between differentially abundant genera and differential metabolites. Rows represent 18 differentially abundant genera and columns represent 30 differential metabolites (VIP > 1.0 and FDR q < 0.05). Colors indicate the Spearman rank correlation coefficient (ρ): red indicates a positive correlation and blue a negative correlation. Asterisks mark significant correlations with Benjamini–Hochberg-corrected q < 0.05. Genera are arranged by enrichment group (enriched in group S at the top and enriched in group H at the bottom), and metabolites are arranged by hierarchical clustering. Genus-level relative abundances were obtained by aggregating ASVs from the UNOISE3 re-analysis, consistent with the data source used in the main text. Metabolite names are database-derived putative annotations; psilocybine and prizidilol are annotated as exogenous (exposome) compounds rather than endogenous metabolites, and correlations involving them should be interpreted with caution. Group H = healthy controls; group S = patients with schizophrenia.

## Discussion

The findings indicate that gut microbial community composition differs between patients with schizophrenia and healthy individuals. In the re-analysis, 21 of the 59 genera examined differed significantly in abundance (FDR q < 0.05), with 4 enriched in the patient group and 17 in the control group. The larger number of control-enriched taxa suggests that schizophrenia here is characterized predominantly by depletion rather than overrepresentation of gut taxa, although the number of significant taxa per group should not be read as a direct measure of ecosystem richness. PCoA and PERMANOVA based on Bray–Curtis distance (pseudo-F = 3.221, R^2^ = 0.053, p = 0.0001) further indicated that the alterations in schizophrenia were not confined to a single taxon but involved a coordinated shift across multiple taxa.

Within this sample, random forest variable importance analysis indicated that Pseudocitrobacter, Hafnia-Obesumbacterium, and Escherichia-Shigella contributed most to between-group discrimination (stratified five-fold cross-validated AUC = 0.989). Because no independent validation set was withheld, neither this ranking nor the discriminative performance should be extrapolated to other cohorts. Genus-level comparisons showed Escherichia-Shigella to be enriched in patients, whereas Faecalibacterium and Agathobacter were more abundant in controls. This pattern is consistent with the phylum-level observation that Proteobacteria increased to 5.2% relative abundance in the patient group; expansion of Proteobacteria is a common feature of gut dysbiosis (41) and is often accompanied by a proinflammatory state. Because Faecalibacterium is a major butyrate producer and Escherichia-Shigella includes proinflammatory species, these compositional differences may have functional consequences. The resolution of 16S sequencing does not allow us to determine whether the detected Escherichia-Shigella sequences represent pathogenic species, however, so this proinflammatory interpretation remains a hypothesis; nor did we measure microbial function or the corresponding metabolites directly, so functional changes cannot be confirmed. Finally, this was a cross-sectional case–control study and can therefore only demonstrate associations among the microbiota, metabolites, and schizophrenia, not causation. The microbial changes could be a consequence of the illness or of confounding factors such as medication, inflammation, and diet, and are not necessarily etiological. Whether these compositional changes are cause or consequence cannot be resolved with cross-sectional data, but they are consistent with the immune and metabolic pathways that link the gut to the central nervous system.

Our β-diversity findings are consistent with many metagenomic and 16S rRNA sequencing studies reporting that the overall structure and taxonomic composition of the gut microbiota in schizophrenia differ from those in healthy individuals. Studies of older adults with schizophrenia in China have likewise shown that patient microbiota cluster separately from those of healthy controls in β-diversity analysis (18), and studies of chronic schizophrenia have reported significant β-diversity differences relative to matched non-psychiatric controls (19).

Findings for α-diversity have been far less consistent. For example, in the Wisket rat model of schizophrenia, community structure differed significantly whereas α-diversity did not (20); a cross-sectional study of first-episode psychosis and of clozapine-treated patients with stable schizophrenia likewise found no difference in α-diversity relative to healthy controls (21). We observed reduced α-diversity in the patient group (Sobs, Chao1, ACE, Shannon, and inverse Simpson indices), adding to this inconsistent picture. Such divergent findings may stem from differences in study populations, disease stages, medication exposure status and sample sizes. Our patients were first-admission inpatients who had received short-term antipsychotic treatment, which contrasts with the absence of an α-diversity decline reported in some first-episode psychosis studies. Possible explanations include residual antipsychotic exposure in our sample (medication duration < 30 days), since antipsychotics can alter the gut microenvironment and reduce microbial diversity, as well as between-study differences in illness severity, geographic population, and diet. In addition, the small sample of this study limits the statistical power of the α-diversity comparisons, which need to be validated in larger samples. Taken together, however, the available evidence indicates that the gut microbial community is imbalanced in patients with schizophrenia.

The microbial features identified here—particularly the enrichment of Escherichia-Shigella and the depletion of Faecalibacterium and Agathobacter—should be regarded as candidate schizophrenia-associated taxa that warrant further investigation; their clinical utility remains to be confirmed in independent cohorts. Future studies should examine whether these microbial features are causally related to clinical outcomes, ideally using larger longitudinal cohorts and mechanistic experiments.

Consistent with the microbial findings, untargeted metabolomic analysis revealed significant metabolomic differences between the two groups. PCA showed clear separation between patients and controls in the space defined by the leading principal components, indicating that overall metabolomic composition differed. This accords with the serum and plasma metabolomic studies of Cui et al. (2020), Liu et al. (2021), and Chen et al. (2020), which reported alterations in amino acid and lipid metabolites in schizophrenia (22–24).

PLS-DA confirmed between-group separation (R^2^X = 0.318, R^2^Y = 0.782, Q^2^ = 0.526), and permutation testing revealed no overfitting (p = 0.005). Applying the criteria of VIP greater than 1.0, fold change above 1.20 or below 0.83, and FDR-corrected q-value less than 0.05, we identified 30 candidate differential metabolites.

According to the KEGG analysis, the differential metabolites belonged mostly to amino acid and lipid metabolism, and in terms of compound classification, carboxylic acids, phospholipids, and amino acids predominated. Because these compounds participate in membrane structure, energy metabolism, and cell signaling, changes in their levels may reflect a metabolic imbalance related to the pathophysiology of schizophrenia.

It should be emphasized that this study identified alterations in amino acid metabolic pathways only through KEGG pathway enrichment; it did not directly quantify key metabolites within these pathways, such as proline, kynurenine, or SCFAs. The mechanistic discussion that follows is therefore inferred from previous studies. Proline is a precursor of neurotransmitters, and disturbed neurotransmitter balance can interfere with glutamatergic transmission and thereby aggravate schizophrenic symptoms; mice lacking PRODH exhibit schizophrenia-like behavioral phenotypes (26). Actual proline levels in our participants will need to be confirmed by targeted measurement.

The tryptophan–kynurenine pathway is another key axis of immune–metabolic dysregulation in schizophrenia identified in previous research: tryptophan is converted to kynurenine by indoleamine 2,3-dioxygenase (IDO) and tryptophan 2,3-dioxygenase (TDO), which in turn gives rise to neurotoxic metabolites such as quinolinic acid (27, 28). If the microbial compositional changes observed here do affect host tryptophan metabolism, this imbalance would be amplified; however, metabolites of this pathway were not detected, so this possibility requires confirmation.

Amino acid dysregulation may also interact with the multisystem changes seen in schizophrenia. Reductions in Faecalibacterium and Roseburia have been linked to disturbed branched-chain amino acid metabolism (29); antipsychotic drugs may aggravate this disturbance through effects on amino acid transporters (30); and metabolites such as kynurenine can modulate the immune system, forming a “microbiota–amino acid–immune” network (31). Amino acid metabolic reprogramming of peripheral T lymphocytes has also been linked to schizophrenia-like phenotypes (25), suggesting that the amino acid alterations observed here may not be confined to the gut lumen. These interactions require confirmation in further mechanistic studies.

This study did not measure short-chain fatty acids (SCFAs) directly, so the following discussion is inferred from the compositional changes described above. SCFA-producing gut taxa (e.g., Faecalibacterium) are reduced in patients with schizophrenia (32–34), consistent with the decreased Faecalibacterium abundance observed here. SCFAs are generated by microbes from dietary fiber and nourish the intestinal epithelium, maintain barrier integrity, and modulate immunity and neuroinflammation (9, 35). A reduction in SCFA-producing genera would therefore be expected to compromise the intestinal barrier, facilitate lipopolysaccharide (LPS) translocation, and promote low-grade inflammation (36). Confirming these inferences will require direct measurement of SCFA levels in this cohort.

Beyond SCFAs, fatty acid metabolism as a whole appears to be markedly disturbed in schizophrenia. A multi-omics comparison found fatty acid metabolites to be more abundant in serum than in feces and to be enriched in the unsaturated fatty acid biosynthesis, arachidonic acid metabolism, and linoleic acid metabolism pathways (37), indicating that this abnormality is systemic rather than confined to the gut. Polyunsaturated fatty acid (PUFA) imbalance is common in patients: decreased erythrocyte membrane PUFA levels have been associated with poorer treatment response (38). Within the n-6 PUFA family, linoleic acid and arachidonic acid metabolism are particularly notable; a prospective cohort study reported a negative association between plasma linoleic acid levels and the risk of schizophrenia, suggesting a possible protective effect (39). Deficiency or disturbed metabolism of omega-3 PUFAs (e.g., EPA and DHA) is associated with cognitive dysfunction and inflammatory states, and clinical trials suggest that omega-3 supplementation may improve psychotic symptoms and cognitive function in patients with schizophrenia (40). Such fatty acid abnormalities can cause neuronal dysfunction by altering membrane fluidity and signal transduction and by modulating inflammatory mediators.

This study has several limitations. First, the sample was small (30 patients and 29 controls with complete sequencing data) and the study was single-center. Because microbiome data show substantial inter-individual variation, small samples are prone to false-positive findings, and no formal sample size estimation or power calculation was performed. To mitigate this, effect sizes and their 95% confidence intervals are reported for the main differential indicators, allowing readers to judge the robustness of the results independently. Second, the cross-sectional case–control design precludes causal inference among gut microbial alterations, metabolomic features, and schizophrenia: the observed microbial changes could be a consequence of the illness or could stem from confounders such as medication, inflammation, and diet. Third, confounding and its statistical control represent the most important methodological limitation of this study. Eligibility required first hospitalization and psychotropic medication for no more than 30 days, but medication-naïve and short-term-medicated patients were not strictly separated, so the cohort still had appreciable antipsychotic exposure; antipsychotics can directly alter the gut microbiota, elevate prolactin, and disturb lipid and amino acid metabolism. Because of the limited sample size, we could neither perform subgroup analyses by medication duration (e.g., < 7 vs. 7–30 days) nor include PRL, E2, and neutrophil count—all of which differed significantly between groups—as covariates in the PERMANOVA and metabolomic analyses, which may have biased the between-group results. The potential impact of antipsychotic exposure is discussed further below in light of drug signals in the untargeted metabolomic data. Fourth, baseline information on BMI, smoking, alcohol consumption, bowel habits, and diet was incompletely recorded and was neither reported nor compared between groups in **Table 1**; key confounders such as diet and bowel habits were therefore neither matched nor statistically adjusted for. In addition, the results of normality testing are not reported separately for each continuous variable. Fifth, because of the taxonomic resolution of 16S rRNA gene sequencing, taxa could be identified only to genus level, and pathogenic could not be distinguished from nonpathogenic species within genera such as Escherichia-Shigella; the functional inferences were not confirmed by shotgun metagenomics or metatranscriptomics. Metabolite identification by untargeted metabolomics and database matching remains putative and requires targeted validation with authentic reference standards; notably, several putatively annotated differential features—including psilocybine and prizidilol—are annotated as exogenous exposome compounds rather than endogenous metabolites, so part of the metabolomic signal may reflect unmeasured xenobiotic exposure. The study also lacked an independent validation cohort and mechanistic experiments. Future work should validate these findings in larger, multi-center longitudinal studies, with particular attention to medication-naïve patients with first-episode schizophrenia.

This drug-related confounding was directly corroborated in our untargeted metabolomic data: among the differential metabolites ranked by VIP, aripiprazole (VIP = 3.95, p = 1.74 × 10^-11^) and its active metabolite dehydroaripiprazole (VIP = 3.14, p = 1.56 × 10^-9^) were detected only in the patient group (aripiprazole: 0/30 in group H vs. 22/30 in group S; dehydroaripiprazole: 0/30 in group H vs. 20/30 in group S) and in no healthy control. Aripiprazole was the most discriminating feature between groups (ranked first by VIP), and its presence reflects psychotropic medication exposure rather than any disease-related biological difference. This confirms that antipsychotics are the most important potential confounder in this study: drugs and their metabolites can simultaneously affect the gut microbiota and the host metabolome, and the overlap of drug and disease effects cannot be ignored when interpreting the “microbiota–metabolite” associations reported here. Because participants were not stratified by medication duration at the time of sampling, we could not statistically separate these drug signals from disease-related metabolic alterations. Distinguishing drug effects from the intrinsic metabolic features of the illness will require future cohorts of medication-naïve first-episode patients or statistical adjustment for medication duration.

Despite these limitations, the design of this study offers additional value. In contrast to most previous schizophrenia studies, which have focused on a single omics layer (9), we integrated three data types within the same cohort: the gut microbiota (16S rRNA gene sequencing), untargeted metabolomics (UHPLC-MS/MS), and serum biochemical indicators (complete blood count, sex hormones, and a comprehensive chemistry panel). Multi-dimensional interpretation can provide information that single-omics studies cannot. Microbiome analysis alone can only establish that microbial composition is altered, without indicating whether this is accompanied by changes in host metabolism or systemic physiology; conversely, metabolomic or serum biochemical analysis alone cannot show how those metabolic changes relate to specific gut microbes. In this cohort, microbial-level findings (reduced α-diversity, β-diversity separation, enrichment of Escherichia-Shigella, and depletion of Faecalibacterium and Agathobacter), metabolic-level findings (alterations in amino acid and lipid metabolic pathways), and serum-level findings (between-group differences in PRL, E2, and neutrophil count) were observed in the same individuals and were mutually consistent. The cross-omics Spearman correlation analysis (**Figure 5**) identified 312 significant correlations (FDR q < 0.05) with clearly group-specific directions. Genera enriched in the patient group were predominantly negatively correlated with differential metabolites, with Escherichia-Shigella as the hub of this negative network, showing significant negative correlations with metabolites such as psilocybine (ρ = −0.628) and PG(a-15:0/5-iso PGF2VI) (ρ = −0.528); SCFA-producing genera enriched in the control group (e.g., Roseburia and Agathobacter) were predominantly positively correlated, including with PG(a-15:0/5-iso PGF2VI) (ρ = +0.58 to +0.64) and prizidilol (ρ = +0.59 to +0.60). This mirror-image pattern—negative correlations for patient-enriched taxa and positive correlations for control-enriched SCFA producers—suggests that the direction of microbial imbalance parallels the direction of host metabolic disturbance, with the proinflammatory microecology represented by Escherichia-Shigella and the barrier-supporting, metabolically favorable profile represented by SCFA-producing taxa lying at opposite extremes. This interpretation is qualified by the fact that two of the strongest contributing features, psilocybine and prizidilol, are annotated as exogenous exposome compounds rather than endogenous metabolites; correlations involving them may reflect undocumented xenobiotic exposure and are therefore regarded as low confidence. At the same time, drug signals such as aripiprazole were significantly negatively correlated with several control-enriched genera (Pseudocitrobacter, Raoultella, and Hafnia-Obesumbacterium), indicating that some of these associations reflect drug exposure specific to the patient group; the cross-omics associations therefore remain exploratory and do not support causal inference. Elevated serum PRL and E2 further suggest that sex hormones participate in microbiota–metabolite regulation. Together, these cross-dimensional consistencies capture the overall “microbiota–metabolite–host” imbalance in schizophrenia better than any single omics indicator. Including serum biochemical indicators can also help to expose confounders: for example, the aripiprazole signals in the patient group indicate that antipsychotic exposure simultaneously affects the microbiota, metabolism, and endocrine function (elevated PRL), providing additional insight for multi-omics study design. Thus, although the multi-omics integration presented here remains exploratory and no formal cross-omics integration model was built, the strategy of parallel multi-dimensional sampling and joint interpretation within a single cohort is more comprehensive than single-omics designs and provides a stronger foundation for integrated “microbiota–metabolite–clinical indicator” mechanistic models in future multi-center longitudinal cohorts. All correlations described above are exploratory, involve no rigorous covariate adjustment or causal inference, and require confirmation by targeted measurements and mechanistic experiments.

The between-group differences in PRL and E2 can also be interpreted in light of bidirectional host–microbiota interactions. Endogenous estrogens such as estradiol are glucuronidated mainly in the liver and excreted into the gut with bile, whereas certain gut microbes (e.g., Escherichia, Bacteroides, and Clostridium) produce β-glucuronidase, which deconjugates conjugated estrogens and allows their reabsorption into the enterohepatic circulation, thereby regulating circulating estrogen levels; the set of microbial genes involved in estrogen metabolism is collectively termed the “estrobolome.” Previous work has described a bidirectional relationship between estrogens and the gut microbiota: estrogens can shape microbial composition, strengthen the intestinal epithelial barrier, and reduce the low-grade inflammation caused by lipopolysaccharide translocation, whereas changes in microbial composition can in turn regulate circulating hormone levels by altering estrogen deconjugation and reabsorption. In this study, enrichment of Escherichia-Shigella (an important source of β-glucuronidase) and depletion of SCFA-producing genera were associated with elevated E2 in patients, suggesting bidirectional interplay between estrogen metabolism and microbial dysbiosis. By contrast, the elevation of prolactin is attributable mainly to dopamine D2 receptor antagonism by antipsychotics, so its association with the microbiota and metabolome more likely reflects drug exposure as a shared upstream driver than an independent microbiota–endocrine mechanism. It should be noted that the between-group differences in E2 and PRL indicate only directional associations: because of the small sample size, no individual-level correlation analysis between sex hormones and microbial abundances or metabolite levels was performed, and the mechanistic associations described above remain speculative, requiring validation in larger cohorts combined with direct measurement of estrogen metabolites and fecal β-glucuronidase activity.

## Conclusion

Compared with healthy individuals, patients with schizophrenia show alterations in gut microbial composition and taxonomic structure as well as in untargeted metabolomic features. Microbiota–metabolite associations emerge against a background of increased Escherichia-Shigella abundance, reduced Faecalibacterium and Agathobacter abundance, and altered lipid and amino acid metabolic pathways. Because the findings are cross-sectional, they cannot establish causality or clinical utility; independent validation in large, multi-center longitudinal cohorts is needed to confirm these findings and to clarify their biological significance.

## Data Availability

The datasets presented in this study can be found in online repositories. The names of the repository/repositories and accession number(s) can be found below: https://www.ncbi.nlm.nih.gov/, PRJNA1401720.

## References

[1] Góralczyk-Bińkowska A Szmajda-Krygier D Kozłowska E . The microbiota-gut-brain axis in psychiatric disorders. Int J Mol Sci. (2022) 23:11245. doi: 10.3390/ijms231911245 36232548 PMC9570195

[2] Asadi A Shadab Mehr N Mohamadi MH Shokri F Heidary M Sadeghifard N . Obesity and gut-microbiota-brain axis: a narrative review. J Clin Lab Anal. (2022) 36:e24420. doi: 10.1002/jcla.24420 35421277 PMC9102524

[3] Gong Y Chen A Zhang G Shen Q Zou L Li J . Cracking brain diseases from gut microbes-mediated metabolites for precise treatment. Int J Biol Sci. (2023) 19:2974–98. doi: 10.7150/ijbs.85259 37416776 PMC10321288

[4] García-Cabrerizo R Carbia C O Riordan KJ Schellekens H Cryan JF . Microbiota-gut-brain axis as a regulator of reward processes. J Neurochem. (2021) 157:1495–524. doi: 10.1111/jnc.15284 33368280

[5] Trzeciak P Herbet M . Role of the intestinal microbiome, intestinal barrier and psychobiotics in depression. Nutrients. (2021) 13:927. doi: 10.3390/nu13030927 33809367 PMC8000572

[6] Guo L Ding Q Li Q Zheng D Guo L Cao X . Anxiety disorders and the gut microbiota: a bibliometric and visual analysis. Front Psychiatry. (2025) 15:1517508. doi: 10.3389/fpsyt.2024.1517508 39902242 PMC11788897

[7] MacKay M Yang BH Dursun SM Baker GB . The gut-brain axis and the microbiome in anxiety disorders, post-traumatic stress disorder and obsessive-compulsive disorder. Curr Neuropharmacol. (2024) 22:866–83. doi: 10.2174/1570159X21666230222092029 36815632 PMC10845093

[8] De Sales-Millán A Aguirre-Garrido JF González-Cervantes RM Velázquez-Aragón JA . Microbiome-gut-mucosal-immune-brain axis and autism spectrum disorder (ASD): a novel proposal of the role of the gut microbiome in ASD aetiology. Behav Sci (Basel). (2023) 13:548. doi: 10.3390/bs13070548 37503995 PMC10376175

[9] Ju S Shin Y Han S Kwon J Choi TG Kang I . The gut-brain axis in schizophrenia: the implications of the gut microbiome and SCFA production. Nutrients. (2023) 15:4391. doi: 10.3390/nu15204391 37892465 PMC10610543

[10] Singh S Sharma P Pal N Kumawat M Shubham S Sarma DK . Impact of environmental pollutants on gut microbiome and mental health via the gut-brain axis. Microorganisms. (2022) 10:1457. doi: 10.3390/microorganisms10071457 35889175 PMC9317668

[11] Doroszkiewicz J Groblewska M Mroczko B . The role of gut microbiota and gut-brain interplay in selected diseases of the central nervous system. Int J Mol Sci. (2021) 22:10028. doi: 10.3390/ijms221810028 34576191 PMC8471822

[12] Ochoa-Repáraz J Ramelow CC Kasper LH . A gut feeling: the importance of the intestinal microbiota in psychiatric disorders. Front Immunol. (2020) 11:510113. doi: 10.3389/fimmu.2020.510113 33193297 PMC7604426

[13] Codoñer-Franch P Gombert M Martínez-Raga J Cenit MC . Circadian disruption and mental health: the chronotherapeutic potential of microbiome-based and dietary strategies. Int J Mol Sci. (2023) 24:7579. doi: 10.3390/ijms24087579 37108739 PMC10146651

[14] El Baassiri MG Raouf Z Badin S Escobosa A Sodhi CP Nasr IW . Dysregulated brain-gut axis in the setting of traumatic brain injury: review of mechanisms and anti-inflammatory pharmacotherapies. J Neuroinflamm. (2024) 21:124. doi: 10.1186/s12974-024-03118-3 38730498 PMC11083845

[15] La Torre D Van Oudenhove L Vanuytsel T Verbeke K . Psychosocial stress-induced intestinal permeability in healthy humans: what is the evidence? Neurobiol Stress. (2023) 27:100579. doi: 10.1016/j.ynstr.2023.100579 37842017 PMC10569989

[16] Bolyen E Rideout JR Dillon MR Bokulich NA Abnet CC Al-Ghalith GA . Reproducible, interactive, scalable and extensible microbiome data science using QIIME 2. Nat Biotechnol. (2019) 37:852–7. doi: 10.1038/s41587-019-0209-9 31341288 PMC7015180

[17] Shrestha P Kim MS Elbasani E Kim JD Oh TJ . Prediction of trehalose-metabolic pathway and comparative analysis of KEGG, MetaCyc, and RAST databases based on complete genome of Variovorax sp. PAMC28711. BMC Genom Data. (2022) 23:4. doi: 10.1186/s12863-021-01020-y 34991451 PMC8734048

[18] Ling Z Jin G Yan X Cheng Y Shao L Song Q . Fecal dysbiosis and immune dysfunction in Chinese elderly patients with schizophrenia: an observational study. Front Cell Infect Microbiol. (2022) 12:886872. doi: 10.3389/fcimb.2022.886872 35719348 PMC9198589

[19] Nguyen TT Kosciolek T Daly RE Vázquez-Baeza Y Swafford A Knight R . Gut microbiome in schizophrenia: altered functional pathways related to immune modulation and atherosclerotic risk. Brain Behav Immun. (2021) 91:245–56. doi: 10.1016/j.bbi.2020.10.003 33098964 PMC8023565

[20] Plesz SB Adlan LG Büki A Makra N Ligeti B Ágg B . Dysbiosis is associated with the behavioral phenotype observed in the triple-hit Wisket rat model of schizophrenia. Prog Neuro-Psychopharmacol Biol Psychiatry. (2025) 137:111276. doi: 10.1016/j.pnpbp.2025.111276 39880276

[21] O’Donnell M Teasdale SB Chua XY Hardman J Wu N Curtis J . The role of the microbiome in the metabolic health of people with schizophrenia and related psychoses: cross-sectional and pre-post lifestyle intervention analyses. Pathogens. (2022) 11:1279. doi: 10.3390/pathogens11111279 36365032 PMC9695516

[22] Cui G Qing Y Hu X Wang P Sun L Yang X . Serum metabolomic profiling based on Fourier transform-ion cyclotron resonance-mass spectrometry: do the dysfunctions of metabolic pathways reveal a universal risk of oxidative stress in schizophrenia? Antioxid Redox Signal. (2020) 33:679–88. doi: 10.1089/ars.2020.8141 32578452

[23] Liu Y Song X Liu X Pu J Gui S Xu S . Alteration of lipids and amino acids in plasma distinguish schizophrenia patients from controls: a targeted metabolomics study. Psychiatry Clin Neurosci. (2021) 75:138–44. doi: 10.1111/pcn.13194 33421228

[24] Chen X Xu J Tang J Dai X Huang H Cao R . Dysregulation of amino acids and lipids metabolism in schizophrenia with violence. BMC Psychiatry. (2020) 20:97. doi: 10.1186/s12888-020-02499-y 32131778 PMC7055102

[25] Wang P Jiang L Hu J Jiang Z Zhang Y Chen C . The amino acid metabolism pathway of peripheral T lymphocytes and ketamine-induced schizophrenia-like phenotype. J Psychiatry Neurosci. (2024) 49:E413–26. doi: 10.1503/jpn-240038 39626901 PMC11633891

[26] Yao Y Jin C Liao Y Huang X Wei Z Zhang Y . Schizophrenia-like behaviors arising from dysregulated proline metabolism are associated with altered neuronal morphology and function in mice with hippocampal PRODH deficiency. Aging Dis. (2024) 15:1952–68. doi: 10.14336/AD.2023.0902 37815900 PMC11272211

[27] Buzásy L Mazák K Balogh B Simon B Vincze A Balogh GT . Physicochemical characterization of kynurenine pathway metabolites. Antioxidants (Basel). (2025) 14:589. doi: 10.3390/antiox14050589 40427471 PMC12108296

[28] Wu X Ao H Wu X Cao Y . Sulfur-containing amino acids and risk of schizophrenia. Schizophr Res. (2023) 262:8–17. doi: 10.1016/j.schres.2023.10.016 37918291

[29] Li J Ma Y Bao Z Gui X Li AN Yang Z . Clostridiales are predominant microbes that mediate psychiatric disorders. J Psychiatr Res. (2020) 130:48–56. doi: 10.1016/j.jpsychires.2020.07.018 32781373

[30] Zhou M Li X Yu W Lin L Liang Y Lu J . MS analysis of the plasma metabolome reveals major changes of amino acid and energy metabolism for early-onset schizophrenia. Am J Transl Res. (2024) 16:6423–34. doi: 10.62347/IDUA6983 39678582 PMC11645623

[31] Fan Y Gao Y Ma Q Yang Z Zhao B He X . Multi-omics analysis reveals aberrant gut-metabolome-immune network in schizophrenia. Front Immunol. (2022) 13:812293. doi: 10.3389/fimmu.2022.812293 35309369 PMC8927969

[32] Li Z Qing Y Cui G Li M Liu T Zeng Y . Shotgun metagenomics reveals abnormal short-chain fatty acid-producing bacteria and glucose and lipid metabolism of the gut microbiota in patients with schizophrenia. Schizophr Res. (2023) 255:59–66. doi: 10.1016/j.schres.2023.03.005 36965360

[33] Karpiński P Żebrowska-Różańska P Kujawa D Łaczmański Ł Samochowiec J Jabłoński M . Gut microbiota alterations in schizophrenia might be related to stress exposure: findings from the machine learning analysis. Psychoneuroendocrinology. (2023) 155:106335. doi: 10.1016/j.psyneuen.2023.106335 37467542

[34] Hawkins B Montgomery M Bokota G Santoyo M Giron E Eltokhi A . Gut microbiota dysbiosis at the interface of neuropsychiatric disorders and their dermatological comorbidities. Gut Microbes. (2025) 17:2574934. doi: 10.1080/19490976.2025.2574934 41277234 PMC12645903

[35] Nshanian M Gruber JJ Geller BS Chleilat F Lancaster SM White SM . Short-chain fatty acid metabolites propionate and butyrate are unique epigenetic regulatory elements linking diet, metabolism and gene expression. Nat Metab. (2025) 7:196–211. doi: 10.1038/s42255-024-01191-9 39789354 PMC11774759

[36] Aghara H Patel M Chadha P Parwani K Chaturvedi R Mandal P . Unraveling the gut-liver-brain axis: microbiome, inflammation, and emerging therapeutic approaches. Mediators Inflammation. (2025) 2025:6733477. doi: 10.1155/mi/6733477 40568349 PMC12197523

[37] Chen L Wang Z Wang Y Jiang H Ding Y Xia Q . Alterations in fatty acid metabolism in patients with schizophrenia in a multi-omics perspective. Schizophr Res. (2025) 279:94–105. doi: 10.1016/j.schres.2025.03.036 40184646

[38] Li N Yang P Tang M Liu Y Guo W Lang B . Reduced erythrocyte membrane polyunsaturated fatty acid levels indicate diminished treatment response in patients with multi- versus first-episode schizophrenia. Schizophr (Heidelb). (2022) 8:7. doi: 10.1038/s41537-022-00214-2 35217671 PMC8881498

[39] Guo X Wu S Jia T Sun Z Yu G Kou C . Plasma fatty acids levels and risk of schizophrenia in the UK Biobank: a prospective cohort study. Psychiatry Res. (2025) 354:116805. doi: 10.1016/j.psychres.2025.116805 41172793

[40] Liu Y Jin X . Fatty acid desaturase 1/2 (FADS1 and FADS2), fatty acid desaturase indices, and their relationships with metabolic syndrome in female adults with first-episode schizophrenia after antipsychotic medications. Psychoneuroendocrinology. (2024) 163:106985. doi: 10.1016/j.psyneuen.2024.106985 38394918

[41] Shin NR Whon TW Bae JW . Proteobacteria: microbial signature of dysbiosis in gut microbiota. Trends Biotechnol. (2015) 33:496–503. doi: 10.1016/j.tibtech.2015.06.011 26210164

