# Supplementary material for: Altered gut microbiota and untargeted metabolomic features in patients with schizophrenia: a case–control study
Source: Front Psychiatry. 2026 Oct 22;17:1981601. doi: 10.3389/fpsyt.2026.1981601 (PMC13736737; doi:10.3389/fpsyt.2026.1981601)
Supplement: Supplementary file 1 [file DataSheet1.pdf]

Supplementary Table S1. Complete effect-size statistics for all 21 differentially abundant genera between the healthy control (H) group and the schizophrenia (S) group (Wilcoxon rank-sum test with Benjamini-Hochberg correction).

Genus	Enriched in	H, median (IQR), %	S, median (IQR), %	log2FC (S vs H)	Wilcoxon U	p	q (BH-adjusted)	Cliff's delta
Hafnia-Obesumbacterium	H	0.022(0.000-0.376)	0.000(0.000-0.000)	-11.40	133	2.00e-07	5.89e-06	-0.694
Pseudocitrobacter	H	0.005(0.000-0.055)	0.000(0.000-0.000)	-18.68	135	7.27e-08	4.29e-06	-0.690
Escherichia-Shigella	S	0.119(0.035-0.200)	2.377(0.664-5.205)	+1.77	716	2.11e-05	3.11e-04	+0.646
Raoultella	H	0.029(0.000-0.102)	0.000(0.000-0.000)	-5.51	161	3.14e-06	6.17e-05	-0.630
Lachnospiraceae NK4A136 group	H	0.338(0.127-0.509)	0.026(0.004-0.116)	-0.94	174	8.01e-05	8.86e-04	-0.599
Lachnospiraceae UCG-008	H	0.301(0.171-0.444)	0.132(0.075-0.175)	-1.06	180	1.14e-04	9.61e-04	-0.586
Citrobacter	H	0.087(0.008-0.774)	0.000(0.000-0.029)	-4.17	183	9.01e-05	8.86e-04	-0.579
[Ruminococcus] gauvreauii group	H	0.579(0.366-0.830)	0.264(0.069-0.443)	-1.08	200	3.77e-04	2.47e-03	-0.540
Agathobacter	H	2.874(1.025-11.077)	0.903(0.347-1.923)	-1.76	212	7.42e-04	4.38e-03	-0.513
Cronobacter	H	0.025(0.000-0.125)	0.000(0.000-0.003)	-4.75	214	3.00e-04	2.21e-03	-0.508
Roseburia	H	2.490(0.702-4.881)	0.728(0.239-1.105)	-1.68	225	1.49e-03	8.00e-03	-0.483
Lachnospiraceae NC2004 group	H	0.617(0.127-1.149)	0.187(0.050-0.266)	-2.09	235	2.49e-03	1.22e-02	-0.460
Faecalibacterium	H	7.275(3.861-11.029)	2.630(1.084-4.701)	-1.26	238	2.89e-03	1.31e-02	-0.453
unassigned	H	1.757(0.848-2.728)	0.864(0.536-1.514)	-0.91	240	3.19e-03	1.34e-02	-0.448
Terrisporobacter	S	0.009(0.001-0.019)	0.097(0.006-1.016)	+4.04	617	5.84e-03	2.17e-02	+0.418
Klebsiella	S	0.012(0.002-0.188)	0.167(0.039-1.042)	+2.40	617	5.88e-03	2.17e-02	+0.418
Enterorhabdus	H	0.280(0.108-0.583)	0.117(0.042-0.210)	-1.32	260	7.94e-03	2.60e-02	-0.403
Christensenellaceae R-7 group	H	0.291(0.020-1.034)	0.021(0.000-0.193)	-1.86	260	7.12e-03	2.47e-02	-0.402
Butyricicoccus	H	0.368(0.148-0.780)	0.132(0.043-0.344)	-0.25	273	1.43e-02	4.16e-02	-0.372
Senegalimassilia	H	0.004(0.000-0.343)	0.000(0.000-0.000)	-3.47	295	1.48e-02	4.16e-02	-0.322
Sarcina	S	0.000(0.000-0.000)	0.000(0.000-0.005)	+11.60	557	8.81e-03	2.73e-02	+0.280

Note. Only the 21 genera with Benjamini–Hochberg-adjusted $q < 0.05$ are listed, sorted by the absolute value of Cliff's delta in descending order. log2FC was calculated from the mean relative abundances between the S and H groups (positive values indicate enrichment in the S group; negative values indicate enrichment in the H group). Percentages are the median (interquartile range) relative abundance. Genera with very low abundance in both groups (e.g., Hafnia-Obesumbacterium, Pseudocitrobacter, Raoultella; median relative abundance approaching 0 in both groups) should be interpreted with caution.
